# Light-driven oxidation of polysaccharides by photosynthetic pigments and a metalloenzyme

**DOI:** 10.1038/ncomms11134

**Published:** 2016-04-04

**Authors:** D. Cannella, K. B. Möllers, N.-U. Frigaard, P. E. Jensen, M. J. Bjerrum, K. S. Johansen, C. Felby

**Affiliations:** 1 Department of Geoscience and Natural Resource Management, University of Copenhagen, Rolighedsvej 23, Frederiksberg C, 1958 Copenhagen, Denmark; 2 Marine Biological Section, Department of Biology, University of Copenhagen, Strandpromenaden 5, 3000 Helsingør, Denmark; 3 Copenhagen Plant Science Centre, Department of Plant and Environmental Sciences, University of Copenhagen, Thorvaldsensvej 40, Frederiksberg C, DK-1871 Copenhagen, Denmark; 4 Department of Chemistry, University of Copenhagen, Universitetsparken 5, 2100 Copenhagen, Denmark; 5 Division of Industrial Biotechnology, Department of Chemical and Biological Engineering, Chalmers University of Technology, Kemivägen 10, SE-412 96 Göteborg, Sweden

## Abstract

Oxidative processes are essential for the degradation of plant biomass. A class of powerful and widely distributed oxidative enzymes, the lytic polysaccharide monooxygenases (LPMOs), oxidize the most recalcitrant polysaccharides and require extracellular electron donors. Here we investigated the effect of using excited photosynthetic pigments as electron donors. LPMOs combined with pigments and reducing agents were exposed to light, which resulted in a never before seen 100-fold increase in catalytic activity. In addition, LPMO substrate specificity was broadened to include both cellulose and hemicellulose. LPMO enzymes and pigment derivatives common in the environment of plant-degrading organisms thus form a highly reactive and stable light-driven system increasing the turnover rate and versatility of LPMOs. This light-driven system may find applications in biotechnology and chemical processing.

## Abstract (web summary)

Lytic polysaccharide monooxygenases are proteins involved in the degradation of plant biomass and are promising biotechnological tools for biofuel production. Here, the authors show that their catalytic activity is significantly boosted when they are combined with photopigments and exposed to light.

## Graphical abstract

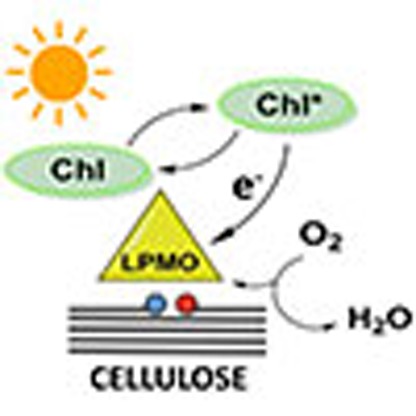

The oxidation of plant biomass by enzyme catalysed reactions is a basic mechanism in the global carbon cycle and of increasing importance and value for industrial applications. The major plant cell wall-derived polymers cellulose, hemicellulose and lignin are oxidized by enzymes, which often are metalloenzymes.

One class of powerful oxidative enzymes is the extracellular copper-based lytic polysaccharide monooxygenases (LPMOs), which are monooxygenases capable of oxidizing the most recalcitrant polysaccharides. When combined with hydrolases there is a strong synergistic effect on biomass degradation^1^,^2^,^3^,^4^. LPMOs are found in fungi, bacteria and viruses, spanning a number of terrestrial and aquatic ecosystems and are classified in the carbohydrate active enzyme database as auxiliary activity (AA) enzymes class 9, 10, 11 and 13 (ref. 5). The enzymes play an essential role, yet not well-understood, in the turnover of organic matter. Some organisms have multiple genes for LPMO enzymes, and especially in plant cell wall-degrading fungi, the AA9 family is highly present with some species containing >30 AA9-encoding genes^5^. All identified substrates are naturally abundant polysaccharides including cellulose, hemicellulose, starch and chitin^6^, in which the glycosidic bonds are oxidized. The products are oxidized oligosaccharides and their non-oxidized counterparts. Specifically for glucans the oxidation of the pyranose ring can take place at either the C1 position producing aldonic acids, or at the C4 position producing 4-ketoaldose (gemdiols). Independently from the C1 and/or C4 type of oxidation, one molecule of dioxygen is needed for the LPMO catalytic cycle: one oxygen atom is inserted in the carbohydrate chain for breaking the glycosidic bond, and the second atom is released from the reaction as part of a water molecule^1^.

The LPMOs have a mononuclear type 2 copper active site, with a t-shaped coordination sphere termed the histidine brace, where two histidines provide three nitrogen ligands, two from N-His and one from the terminal amine. Moreover, LPMOs require an extracellular electron donor to complete their catalytic cycle, and both proteins and plant-derived molecules have been found to serve as donors^1^,^7^,^8^. However, the specific types of electron donors, as well as the mechanism of electron transfer are still unresolved. Ascorbic acid has often been used as electron donor experimentally, and is also present in non-lignified plant biomass, but other donors such as lignin have been described^9^,^10^,^11^. In addition, the enzyme cellobiose dehydrogenase (CDH) functions as a donor with the electron transfer presumably taking place from the haem b-containing cytochrome domain of CDH^8^,^12^,^13^. However, as CDH is absent in many LPMO-expressing organisms, it is not an absolute prerequisite for all LPMO activity in nature^14^.

The apparent promiscuity of LPMOs with respect to the source of electrons, prompted us to investigate the effect of one of the most potent electron donors in nature namely chlorophylls, which are essential components of the photosynthetic light-harvesting and reaction centre complexes. The energy of electromagnetically excited chlorophyll *a* is 1.85 eV and 1.33 eV for the singlet and triplet state, which corresponds to reduction potentials of −1.07 V and −0.55 V for the oxidized chlorophyll to excited state chlorophyll in solution, respectively^15^. Thus, a simple photosystem was set up for light-induced electron transfer consisting of thylakoids or chlorophyllin as electron-donating pigments, an electron accepting LPMO enzyme with an estimated redox potential of +0.22 V (ref. 15) and a reductant ascorbic acid, reducing the oxidized pigment. The redox potential of ascorbic acid at pH 7 is +0.35 V in its radical form and +0.06 V when oxidized to dehydroascorbate. To excite the pigments, the samples were exposed to light of different wavelengths and in different time intervals.

Previous examples of light-driven enzymatic processes are hybrid systems of carbon monoxide dehydrogenase molecules with CdS nanocrystals for light-driven reduction of CO_2_ (ref. 16) and a hydrogenase and TiO_2_ nanoparticle for light-driven H_2_ generation^17^. However, here we combine abundant biological parts: chlorophyll pigments and LPMO enzymes, and report that the catalytic activity of LPMOs can be driven by light and in some cases increased up to 100-fold by light-induced electron transfer. Using both sunlight and LED light sources of different colours, isolated thylakoids from photosynthetic organisms, as well as the chlorophyll derivative chlorophyllin increase the reactivity of three different LPMOs. In addition, we show how different reductants can be applied and suggest how light-induced oxidation may be an active mechanism in the global carbon cycle.

## Results

### Light boosted LPMO activity: oligosaccharide products

The activity of the LPMOs was detected in an assay using phosphoric acid-swollen cellulose (PASC) as substrate, and the released oligomeric products were detected and quantified by high-performance anion exchange chromatography (HPAEC) after incubation. The gluconic acid on the C1 oxidized oligosaccharides was quantified and used as a measure of enzyme activity. The assay for quantification was based on a complete hydrolysis of the oxidized PASC with a cellulase mixture and subsequent detection of gluconic acid by HPAEC.

**Figure 1:**
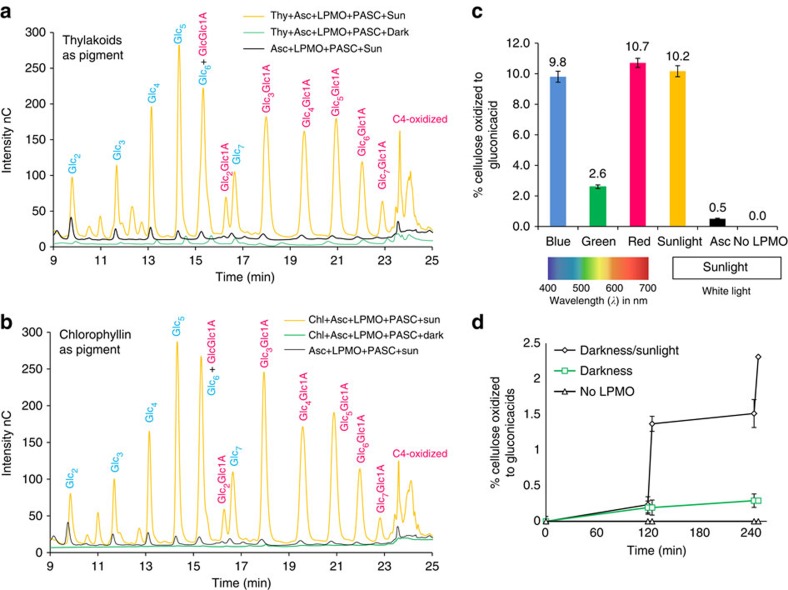
Cellulose oxidation by *Tt*LPMO9E combined with light-induced electron transfer. The released oligosaccharides from PASC were measured by HPAEC showing non-oxidized (blue) and oxidized (magenta) reaction products. Each chromatogram is average of three replicate experiments. (**a**) Thy+Asc+LPMO+PASC+Sun: Thylakoids, ascorbic acid and LPMO with PASC in sunlight for 3 h (150–200 μmol photons s^−1^ m^−1^), yellow line. Control 1 Thy+Asc+LPMO+PASC+Dark: same conditions but in darkness, green line. Control 2: Asc+LPMO+PASC+Sun: same conditions but without thylakoids, black line. (**b**) Same experiments as in **a** but with chlorophyllin (Chl) as pigment. Peak annotations of native oligosaccharides (blue) were done using the pure compounds as standard: Glc_2_, cellobiose; Glc_3_, cellotriose; Glc_4_, cellotetraose; Glc_5_, cellopentaose; Glc_6_, cellohexaose; Glc_7_, celloheptaose; oxidized oligosaccharides (magenta) were assigned by comparing with literature chromatograms performed with identical separation conditions as done by Westereng^37^: GlcGlc1A, cellobionic acid; Glc_2_Glc1A, cellotrionic acid; Glc_3_Glc1A, cellotetraonic acid; Glc_4_Glc1A, cellopentaoinic acid; Glc_5_Glc1A, cellohexaoinic acid; Glc_6_Glc1A, celloeptaonic acid; Glc_7_Glc1A, cellooctaonic acid. On the *y*-axis is reported the intensity of the signal in nC (nano Coulomb) without further adjustments. (**c**) PASC oxidation with LPMO (*Tt*AA9E), chlorophyllin and ascorbic acid in response to blue, green, red light (150 μmol photons s^−1^ m^−1^) and sunlight (150–200 μmol photons s^−1^ m^−1^). Oxidation was measured with HPAEC quantifying the gluconic acid (oxidation of the C1 position at the pyranose ring). Asc: PASC and ascorbic acid in sunlight (black bar). No LPMO: PASC, chlorophyllin and ascorbic acid in sunlight. (**d**) Chlorophyllin, ascorbic acid, LPMO and PASC incubation was run for two cycles of darkness for 2 h and sunlight for 5 min, or always in darkness (4 h 10 min), black line with diamonds and green light with square, respectively. Negative control experiment missing LPMO was run in parallel with the darkness/sunlight cycles. The percentage of oxidized cellulose was measured by quantification of gluconic acid formed (C1 oxidation).

Initially, PASC was exposed to LPMO from *Thielavia terrestris* and suspended thylakoid membranes from a cyanobacterium (*Synechococcus* sp. PCC 7002) in the presence of ascorbic acid and sunlight. The light response resulted in a 20-fold increase in release of oligosaccharides as compared with incubation in darkness or incubation under conventional conditions with ascorbic acid and light (without the thylakoids, Fig. 1a). Replacing the cyanobacterial thylakoids with membranes prepared from a higher plant (*Arabidopsis thaliana)* produced similar results (Supplementary Fig. 1).

As LPMOs require dioxygen, we wanted to rule out that dioxygen produced by the thylakoid membranes was the cause of the observed activity enhancement, and therefore the water-soluble chlorophyll derivative chlorophyllin was tested in the same assay. This resulted in an equally pronounced light-driven accumulation of oxidized products (Fig. 1b), showing that chlorophyll pigments on light excitation transfer electrons to the LPMO, regardless of whether the chlorophyll pigment is membrane-bound (thylakoids) or soluble (chlorophyllin).

The light-driven oxidation was expanded to experiments with two LPMOs from *Thermobifida fusca* and *Thermoascus aurantiacus* (Supplementary Figs 2 and 3). For all enzymes tested, both C1 and C4 oxidation increased in the light-driven system. Not only the swollen PASC cellulose but also crystalline cellulose in the form of Avicel was tested as a substrate, and it was similarly oxidized by the light-driven system (Supplementary Fig. 4).

To investigate the light response of the combined pigments and LPMO-catalysed cellulose oxidation, experiments were performed using blue, red, green and sunlight with the same range of intensity. In accordance with the absorption spectra of chlorophyll pigments: sunlight, blue and red light induced the highest levels of cellulose oxidation (Fig. 1c). A light response experiment with alternating darkness and sunlight further proved that light activated the oxidation of cellulose (Fig. 1d).

The level of oxidized products from a 3-h light-induced reaction (Fig. 1c) measured by the amount of glucose units oxidized to gluconic acid, shows that ∼10% of the cellulose was oxidized using photo-excited pigments and sunlight, compared with 0.5% without pigments and only ascorbic acid and sunlight. No products were detected without the enzyme (Fig. 1c,d). The levels of cellulose oxidation catalysed by light-induced electron transfer and LPMOs are to the best of our knowledge higher than any previously reported result^1^,^9^.

Control experiments were performed to investigate the light response of thylakoids and chlorophyllin when incubated with LPMO and without any reductant: the pigments alone were able to donate electrons to LPMO but were subject to photobleaching and degradation (Supplementary Figs 5 and 6 and Supplementary Discussion). Non-enzymatic reactions from light-induced reactive oxygen species, which potentially can break down cellulose, were also investigated. This was done by combining the pigment preparations and ascorbic acid, but without LPMO enzyme added. These experiments did not result in oxidation of cellulose and confirmed that the applied pigments or ascorbic acid caused cellulose oxidation only when combined with LPMO (Fig. 1c,d, and Supplementary Fig. 7).

### Oxygen consumption

**Figure 2:**
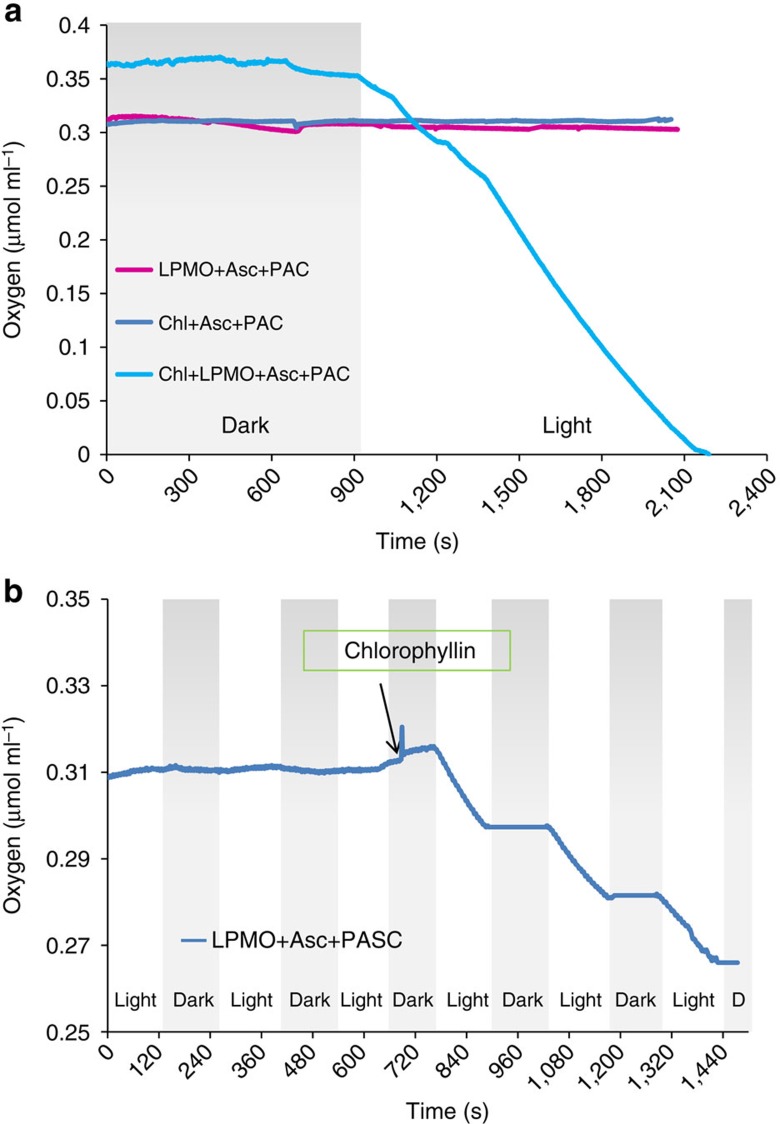
Oxygen consumption during cellulose oxidation with light or in darkness. (**a**) Oxygen consumption with different combinations of the photosystem: blue line shows chlorophyllin, ascorbic acid and PASC (without *T. terrestris* LPMO); magenta line shows *T. terrestris* LPMO, ascorbic acid and PASC; the turquoise line shows the whole photosystem with chlorophyllin, ascorbic acid, *T. terrestris* LPMO and PASC. Light is turned on at 900 s. (**b**) Alternating cycles of light and darkness: at the beginning of the incubation only *T. terrestris* LPMO, ascorbic acid and PASC are present, then chlorophyllin is added after 700 s.

As LPMOs require oxygen for their catalytic cycle, oxygen measurements were used to monitor the reaction catalysed by the *T. terrestris* LPMO. The results show that oxygen is consumed by the photosystem in a light-dependent manner. In Fig. 2a, the oxygen consumption is shown during incubation in light or darkness: from 0 to 900 s the reactions were carried out in darkness, while from 900 to 2400, s white light was applied. Without the enzyme (blue line) there was no oxygen consumption observed. Without chlorophyllin but with LPMO and ascorbic acid (magenta line) the oxygen consumption was very low, and there was no response to light.

For the complete photosystem composed of reductant, pigment and enzyme (turquoise line) there was the same oxygen consumption in darkness as seen for the incubation without chlorophyllin (magenta line), but when the light was switched on (at 900 s) a rapid consumption of oxygen was observed. The oxygen consumption correlated with the release of gluconic acid. At the end of the reaction after 2,400 s, 0.7% of the PASC cellulose was oxidized at the C1 positions to gluconic acid, corresponding to a stoichiometric ratio of 1:0.8 between dioxygen consumption and C1 product formation. As the C4 oxidation products are not included, it is reasonable to assume a 1:1 ratio between dioxygen and oxidation products. Without the pigment (magenta line), the oxidation products were below the detection limit.

The photosystem reactivity could be controlled by switching the light on and off (Fig. 2b). At the start of the experiment, only LPMO, ascorbic acid and PASC were present. After five cycles of light and darkness there was no sign of oxygen consumption. After 700 s, chlorophyllin was added, followed by a clear oxygen consumption in light and no oxygen consumption in darkness (Fig. 2b).

### Non-cellulosic substrate and lignin as reductants

**Figure 3:**
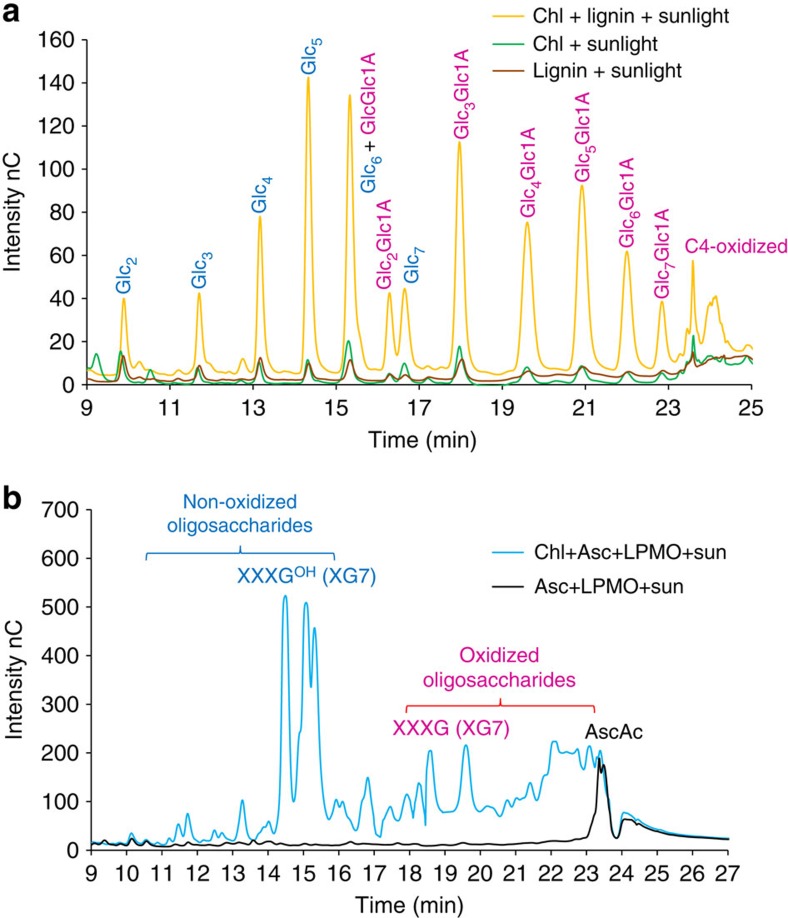
HPAEC analysis of light-induced polysaccharide oxidation with *Tt*LPMO9E. (**a**) Lignin as reductant: LPMO oxidative activity on cellulose (PASC) when incubated with chlorophyllin and organosolv lignin as reductant in 3 h of sunlight. Controls with only chlorophyllin or organosolv lignin in sunlight are included. Each chromatogram is average of three replicate experiments. For details on peak annotation see Fig. 1 or Supplementary Fig. 1. (**b**) Xyloglucan as substrate: LPMO oxidative activity on xyloglucan (1% w/w) when incubated with chlorophyllin and ascorbic acid and exposed to 3 h of sunlight. Pronounced is the presence of oxidized species detectable from minute 17 to 24. A control experiment with LPMO and ascorbic acid shows no degradation of the xyloglucan. The xyloglucan is a heteropolymer of xylose, glucose, galactose and arabinose. The division of peaks in oxidized and non-oxidized is derived from the model heptamer xylooligosaccharide XXXG^OH^ in its reduced form made of four units of glucose of which three are substituted with one xylose. While XXXG is the same, but oxidized in the reducing end of the last subunit in the glucan backbone^19^,^20^.

Ascorbic acid is a naturally available reductant for pigments only in non-lignified plant tissue. To look for alternative natural reductants, we replaced ascorbic acid with extracted lignin. Indeed, insoluble lignin extracted from wheat straw could replace ascorbic acid in the light-driven assay with chlorophyllin (Fig. 3a). This points to how light-induced electron transfer may also be functional in degradation of lignified biomass. The functionality of lignin as a multiple electron donor to the pigments may be ascribed to its electrons in de-localized and overlapping *π*-orbitals^18^.

To our surprise, the specificity of the *T. terrestris* LPMO changed when used in the light-induced electron-transfer assays. This particular LPMO is thought to be strictly cellulose specific, but when combined with light-induced electron transfer from pigments, the enzyme also oxidized the hemicellulose xyloglucan (Fig. 3b). Oxidation of both cellulose and hemicellulose has been reported for LPMOs from *Neurospora crassa*^19^ and from *Podospora anserine*^20^. The degradation pattern of xyloglucan in Fig. 3b is similar to a xyloglucan degradation pattern previously reported^20^.

## Discussion

**Figure 4:**
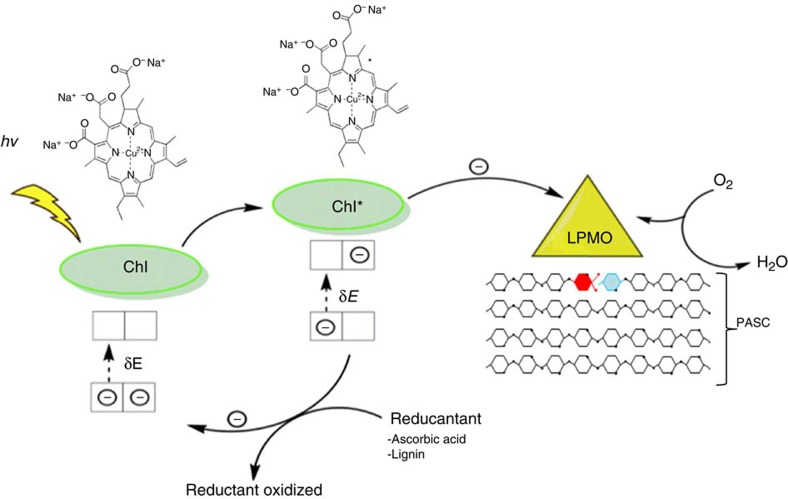
Proposed mechanism for light-induced electron transfer to LPMO. Light excites the pigment, which in its excited state transfers an electron to the LPMO enzyme. The excited electron reduces the copper in the LPMO-active site, which then activates oxygen and oxidizes the polysaccharide. The oxidized pigment is reduced by acquisition of an electron from a reductant such as ascorbic acid or lignin, thus now available for a new cycle of excitation. During the monooxygenase reaction of LPMO, dioxygen is split, and one oxygen atom is incorporated into the substrate, and the other is reduced to water. The oxidation of a PASC cellulose chain is shown as a red monomer of glucose oxidized in the C1 position to an aldonic acid oligosaccharide, whereas the blue monomer will be released as a non-oxidized oligosaccharide.

The light-induced boosting of PASC oxidation presented here clearly shows the pivotal role of the LPMO enzyme for the light-induced oxidation of carbohydrates. We suggest a model in which light excitation of the pigments generates a strong reductant that efficiently reduces the copper ion in the LPMO enzyme by donating an electron as shown in Fig. 4. Subsequently the oxidized form of the pigments is reduced by a reductant with lower redox potential, in this case ascorbic acid or lignin.

The initial observation of light-induced boosting of PASC oxidation raised the question if non-enzymatic Fenton-reactions were induced by light. Exposure of chlorophyllin or thylakoid preparations, which contain magnesium, copper and zinc, could by themselves generate hydroxyl radicals, that potentially are powerful oxidizers^21^. However, the extensive control experiments examining all the system components revealed no formation of oxidation products from such reactions (Supplementary Fig. 7), also there was no detectable oxygen consumption with an enzyme-free control (Fig. 2a).

Not only the level of oxidized products, but also the reactivity increased with light-induced electron transfer. Using ascorbic acid or CDH as electron donors, an end-level of ∼2% oxidized cellulose is reached after 24–36 h (refs 9, 20). With the light-induced electron-transfer system, the level of LPMO-oxidized cellulose was 2.3% after two cycles of 5 min (Fig. 1d, Supplementary Fig. 8 and Supplementary Discussion). It is thus possible to achieve the same level of oxidation in less than a hundredth of the time required without light-induced electron transfer. These data are also supported by the LPMO turnover frequency determined to be 0.25 s^−1^ based on the oxygen consumption when incubated with the photosystem at 25 °C (Fig. 2a) and 0.55 s^−1^ at 50 °C based on the accumulation of oxidized product after two cycles of 5 min light exposure (Fig. 1d). Previously published turnover values for a *N. crassa* LPMO by measuring reducing ends of oxidized PASC at 50 °C were 0.01–0.04 s^−1^ (ref. 22).

Based on our results, we conclude that the catalytic activity for LPMOs combined with the photosystem for light-induced electron transfer results in up to 2 orders of magnitude higher level of catalytic activity than previously reported.

The results presented support the proposed mechanism and synergy of light-induced electron donation for the LPMOs (Fig. 4). But how are the electrons donated to the active site, directly or via long-range transfer through the protein? Tan *et al*.^12^ suggested a direct electron transfer to the LPMO-active site from CDH via a haem-binding cytochrome complex. Another possibility is through protein long-range electron transfer. This allows the enzyme to be adsorbed to its substrate during the electron transfer, and a possible pathway for electron transfer from CDH has been suggested^3^. In the LPMO AA9 family we found, using molecular modelling, other possible electron-transfer pathways from the surface to the active site, potentially capable of transferring electrons from the excited pigments. Specifically for the *Ta*LPMO9A a putative pathway goes from a histidine (His87) surface ligand through 12 covalent bonds to the Cu(II), equivalent to a tunnelling length of 16.8 Å with a calculated pathway coupling decay value of 2.2 × 10^−3^. The histidine is situated on the side of LPMO and thus free of cellulose that is binding to the LPMO during catalysis. Similar pathways were located in other AA9 LPMOs all located very much in the same position but with different ligands at the surface (Supplementary Fig. 9). The exact mechanism for the suggested electron transfer is so far unresolved, and the existence of parallel paths cannot be ruled out.

As the light-induced electron transfer reported caused a tremendous increase in products, as well as a change in enzyme specificity, the energy of the transferred electrons seems to alter the cycle or state of the active site. As suggested above, the high-energy photoelectron from pigments could be donated by long-range electron transfer^23^. Possible targets could be [LPMOCu(II)OO·] and/or [LPMO–Cu(II)-OOH] (−OOH=deprotonated hydrogen peroxide), intermediates in the catalytic cycle of LPMO proposed by Philips *et al*.^8^, and later studied by quantum mechanical calculations^24^,^25^. The products from the latter reduction could via a Fenton-type reaction form OH·, OH–, Cu(II) (or alternatively Cu(III) and 2 OH–) capable of performing the observed polysaccharide oxidation^26^,^27^,^28^.

Abiotic light-driven degradation of biomass is well-described^29^, here we have identified a biotic mechanism for light-driven degradation. Components common in the natural environments of plant-degrading organisms (that is, chlorophylls and breakdown products hereof, other porphyrins and lignin) can together with LPMOs form a highly reactive system capable of using light energy to boost the degradation of plant cell walls. In light-exposed environments, microorganisms can use electromagnetic radiation to increase the release of glucose and other metabolizable carbohydrates. Our results show that LPMOs can use photo-excited electrons for their catalytic cycle, and the wide distribution of LPMOs in nature suggests that biotic photodegradation of cell wall materials can be a widespread mechanism in different ecosystems. However, the findings of this work need to be extended to prove such mechanisms, and further work may find inspiration from the observations of light-enhanced enzymatic activity in forest floor litter^30^.

The observed light-driven enzymatic oxidation performed by LPMO consumes oxygen, oxidizes carbon and produces water. Looking ahead, this process may find applications in technology areas for chemical processing such as conversion of recalcitrant biomass to fuels and chemicals. We speculate that the light-induced oxidation can be applied in a broader application scheme for oxidation of C–H bonds.

## Methods

### Enzymes

Purified *T. terrestris* LPMO (*Tt*LPMO9E, previously *Tt*GH61E) and *T. auranticus* (*Ta*LPMO9A) were donated from Novozymes A/S (Denmark). The enzymes are produced by expression in a host organism and subsequently purified. *T. fusca* AA10 (TfLPMO10A) cloned and expressed in *Escherichia coli* was purchased from Nzytech Ltd (Portugal). All LPMOs were free of any residual cellulase or hemicellulose activities. Commercial cellulase mixtures Celluclast 1.5l and Novozym 188 were obtained from Novozymes A/S. The Celluclast 1.5l mixture had a protein content of 127 mg g^−1^, containing 62 filter paper units (FPU) per g, and a β-glucosidase activity of 15 U per g. Novozym 188 had a protein content of 220 mg g^−1^, containing a β-glucosidase activity of 231 U per g.

### Chemicals, materials and substrates

Ascorbic acid was obtained from Sigma-Aldrich. Stock solutions of 100 mM were made in water and kept at −20 °C in the dark. Avicel microcrystalline cellulose was obtained from Sigma-Aldrich. Xyloglucan product code P-XYGLN was obtained from Megazymes Ltd, Ireland. Chlorophyllin product code C-100.000-WS-P produced by extraction of *Festuca arundinacae* was obtained from Chr. Hansen, Hørsholm, Denmark.

### Preparation of PASC microcrystalline cellulose substrate

Avicel (microcrystalline cellulose, Sigma-Aldrich PH101) was swollen with phosphoric acid to generate PASC as described by Wood^31^ with a few modifications: 4 g of Avicel were suspended in 100 ml of phosphoric acid (85% w/v) at 40 °C and magnetically stirred for 1 h. The mixture was poured into 1.9 l of water at 40 °C and stirred for 1 h. The suspension was washed four times with 2 l H_2_O (MilliQ-quality), two times with 2 l of a 1% NaHCO_3_ solution to reduce acidity, and then three additional times with 2 l H_2_O and stored at 4 °C. The final cellulose content of the PASC suspension was determined by enzymatic hydrolysis (24 h, 50 °C), with an enzymatic dosage of 75 FPU per g of cellulose, using Celluclast 1.5L cellulolytic enzymes and Novozym 188 in a 5:1 ratio; the released glucose was measured according to the protocol described in section ‘Enzymes' giving an estimated cellulose content of 1.5% w/v. The average degree of polymerization (DP) of Avicel-derived PASC was determined by measuring the total number of reducing ends^32^ and comparing this with the total amount of monomeric glucose, giving a DP of 52.

### Organosolv lignin extraction

The lignin fraction was prepared from wheat straw (*Triticum aestivum* L.). The straw was ball milled for 20 min and the cellulose and hemicellulose fractions were removed by hydrolytic enzymes (Celluclast 1.5L and, Novozym 188 in a 5:1 v/v) at a final dosage of 75 FPU per g of dry lignocellulose substrate for 144 h at 50 °C followed by washing with MilliQ water at a ratio of 1 l for 1 g of dry material. The amount of residual carbohydrates was <2% in the final material. Finally, the residual material was suspended in an aqueous ethanol solution (50:50 water/ethanol) at a 5:1 liquid/solid ratio and heated at 220 °C in a 1 l Parr reactor for 80 min. After heating, the lignin residue was filtered at 75 °C. Solubilized lignin was precipitated by adding water at three times the original amount and recovered by filtration. The insoluble lignin fraction was dried at 40 °C and ground with a pestle and mortar.

### Preparation of chlorophyllin

A stock solution of 12% w/v (166 mM) chlorophyllin was prepared by dissolving the powder in water (MilliQ-quality). The chlorophyllin was kept in darkness while stored at 4 °C. Prior to each experiment, an aliquot of the stock solution was incubated in darkness for 2 h at room temperature.

### Preparation of cyanobacterial thylakoid suspensions

Thylakoid suspensions, containing light-harvesting antennae (phycobilisomes) and thylakoid membranes, were prepared from the cyanobacterium *Synechococcus* sp. PCC 7002 (referred to as *Synechococcus*) grown in medium A containing 2 g NaNO_3_ per l as previously described^33^. *Synechococcus* cells were harvested in 50 ml volumes by centrifugation (5,000*g* for 5 min) and subsequently resuspended in 1 ml thylakoid washing buffer (pH 6.35) as described^34^. The resolved pellet was transferred to a microfuge tube containing 500 μl glass beads (glass beads for cell disruption, 0.1–0.25 mm diameter, Retsch Technology GMBH, Haan, Germany) followed by cell disruptive sonication (Amplitude 50, 3 min processing time, 5 s on/off cycle). The cell extract was then centrifuged (12,000*g*, 4 °C, 20 min) and the pellet, containing unbroken cells and cell walls, was discarded. An additional centrifugation (40,000*g*, 4 °C, 30 min) separated the light-harvesting antennae (phycobilisomes, supernatant) from the thylakoid membranes (pellet). Absorption spectra of the thylakoid suspensions in the supernatant were performed with a UV1800 spectrophotometer (Shimadzu, Kyoto, Japan) and the chlorophyll *a* content was calculated as described^35^. The thylakoid suspensions were then used to conduct the light-induced electron-transfer experiments with an average concentration of 0.21±0.06 mg Chl per ml. Putative contaminations derived from cytoplasmic membranes in the thylakoid suspension have no impact on light-induced electron transfer to LPMOs, this was tested with disrupted *E. coli* K-12 cells

### Preparation of plant thylakoid membranes

Plant thylakoid membranes were extracted from *A. thaliana* (L.) Heynh. Ecotype Columbia. Plants were grown in compost in a controlled environment *Arabidopsis* chambers (Percival AR-60 I, Boone, IA) at a photosynthetic flux of 130–150 μmol of photons per m^2^ per s, 20 °C and 70% humidity. Leaves from ∼25 plants were pooled and homogenized using a blender fitted with razor blades in ice cold buffer containing 20 mM Tricine (pH 7.5), 10 mM NaCl, 5 mM MgCl_2_, 0.4 M sucrose, 5 mg ml^−1^ bovine serum albumin (BSA) and 100 mM sodium ascorbate. The homogenate was immediately filtered through two layers of nylon mesh (31 μm pore size), after which the filtrate was centrifuged (6,000*g*, 4 °C, 15 min). The pellet was resuspended in 5 mM Tricine (pH 7.9) to lyse the chloroplasts. Following lysis, the thylakoids were collected by centrifugation (17,200*g*, 4 °C, 10 min). The pellet was resuspended in a small volume of homogenization buffer without ascorbate and BSA but with 20% glycerol (v/v). Total chlorophyll (Chl) and Chl *a*/*b* ratio were determined in 80% acetone according to the study by Lichtenthaler^36^. The final concentration of the thylakoid membranes applied in the light-induced electron-transfer experiments was 3.57 mg Chl per ml (Chl a/b ratio=3.0).

### Elemental analysis inductively coupled plasma (ICP)

Chlorophyllin and thylakoid suspensions were analysed for the presence of metals on an Aurora Elite ICP-MS system from Bruker. Samples of chlorophyllin 12% (w/w) and extracted thylakoid suspensions 2% (w/w) were diluted to 500–1,000 p.p.b. dry matter in 1% nitric acid. The samples were analysed for ^24^Mg, ^25^Mg, ^26^Mg ^63^Cu, ^65^Cu, ^66^Zn, ^67^Zn and ^68^Zn. Chlorophyllin contained 0.41% Mg, 2.0% Cu and 0.05% Zn relative to total dry matter. Extracted thylakoid suspensions contained 1.8% Mg, 0.007% Cu and 0.0007% Zn relative to total dry matter.

### Quantification of glucose and cellobiose by HPLC

The quantification of D-glucose and D-cellobiose was done using an Ultimate 3000 high-performance liquid chromatography (HPLC, Dionex, Germering, Germany) equipped with refractive index detector (Shodex, Japan) and ultraviolet detector at 210 nm (Dionex). The separation was performed in a Phenomenex Rezex ROA column at 80 °C with 5 mM H_2_SO_4_ as eluent at a flow rate of 0.8 ml min^−1^.

### Measurement of oligosaccharide and gluconic acid by HPAEC

The samples were prepared as follows: 200 μl were centrifuged at 14,000*g* for 2 min and 100 μl of the supernatant was inserted in the HPLC conical vial, without any further light exposure (wrapped in aluminium foil). HPAEC was run on an ICS5000 system (two different machines available in the laboratory), equipped with a PAD detector (Dionex, Sunnyvale, CA, USA) with a CarboPac PA1 column (2 × 50 mm guard column followed by a 2 × 250 mm analytical column) and operated at a flow of 0.25 ml min^−1^, at 30 °C. Chromatographic analysis of aldonic acids separation was conducted as described by Westering *et al*.^37^. The elution involved a linear gradient from 100% *A*:0% *B* to 90% *A*:10% *B* (10 min), followed by an exponential gradient to 70% *A*:30% *B* (15 min) and last an exponential gradient to 100% *B* (5 min). After that a linear gradient was applied for 15 min at the initial conditions 100% *A*:0% *B* (eluent *A*=0.1 M NaOH, *B*=0.1 M NaOH and 1 M NaOAc)^37^. The peaks of the cellulose oligomers in the chromatograms are assigned according to Westereng *et al*.^37^. The chromatograms shown in the main paper, and in Supplementary Figs 1–7, are average of three independent experiments.

### Quantification of cellulose oxidation for TtLPMO9E

To quantify the total oxidation of cellulose (as shown in Fig. 1c), the residual PASC together with the supernatant containing oligosaccharides were digested with a commercial cellulase mixture, Celluclast supplemented with beta-glucosidase Novozym 188 in a 5:1 v/v ratio (both lacking LPMO activity) and applied based on Celluclast at 75 f.p.u. per g cellulose for 5 h at 50 °C in darkness. These conditions ensured complete hydrolysation of the PASC material. The LPMO reaction was stopped prior to cellulases treatment by boiling the vials/samples for 10 min. The hydrolysates were then analysed by HPLC to quantify the D-glucose and in the HPAEC for quantification of gluconic acid (marker of C1 oxidizing activity of LPMO). A minor C4 component (annotated C4-oxidized after minute 24) appeared in the oxidized products; it was impossible to quantify the amount of the monomeric 4-keto aldose sugar (C4-oxidized glucose) as due to tautomerization there are no standards available.

### Standard conditions for enzymatic reactions and LIET

The light-induced electron-transfer (LIET) system was composed of PASC incubated with an LPMO enzyme, a light-absorbing component (chlorophyllin, cyanobacterial thylakoid suspensions or plant thylakoid membranes) and a reductant, that is, ascorbic acid. All experiments presented in the paper, as well as in the Supplementary Information were conducted in triplicates, and repeated on two different ICS5000 HAPEC systems available in the laboratory.

The standard reaction mixture composition for PASC oxidation reactions was: 0.75% w/v PASC, 2 mM ascorbic acid, 100 mM of citrate-phosphate buffer (pH 6.3) and 0.05 mg ml^−1^ LPMO in a 200 μl reaction volume. For the *Tt*LPMO9E with an estimated molecular weight of 22.5 KDa this equals 2.2 nmol of added enzyme. Chlorophyllin was added in a 1:100 dilution out of a 12% stock solution thus a final concentration of 1.6 mM. Freshly prepared cyanobacterial thylakoid suspensions and plant thylakoid membranes were diluted into the reaction mixture 1:2 and 1:20, respectively. The described composition applies for reactions using the AA9 enzymes (*Tt*LPMO9E and *Ta*LPMO9A) and is referred as standard conditions in the rest of the Methods section. For reactions with the AA10 enzyme (*Tf*LPMO10A), a phosphate buffer at pH 7.8 (20 mM) was used and 0.20 mg ml^−1^ of the enzyme preparation was used.

The standard conditions for the light-induced electron-transfer system for cellulose oxidation are defined as: 3 h incubation time, irradiance intensity for sunlight was 150–200 μmol photons per m^2^ per s, for blue, red and green light sources 150 μmol of photons per m^2^ per s (custom made LEDs for blue light 440 nm, red light 625 nm and green light 528 nm, with a spectral width 18 nm, 18 nm and 33 nm respectively). The irradiance intensities reported were measured at the bottom of the closed 2 ml Eppendorf test tubes, and the light probe (a Spherical Micro Quantum Sensor US-SQS/Lund) was submerged into the reaction mixture to account for light-scattering effects caused by PASC cellulose or other insoluble particles. Incubations were carried out in 2 ml Eppendorf polypropylene microfuge tubes in a thermomixer at 1,000 r.p.m. and 50 °C (Eppendorf thermomixer, Eppendorf, Hamburg, Germany). The thermomixer was modified to carry a transparent microfuge tube rack allowing light penetration through the walls of the microfuge tubes. All experimental preparations were carried out in dim green light at 0 μmol of photons per m^2^ per s (Phillips TL-D 36 W colour green Lumen 3600, 540 nm). The degree of PASC oxidation was determined by quantification of oxidized glucose units being the gluconic acid. Optimization of reaction conditions in terms of time of incubation and reactant dosages are shown in Supplementary Fig. 8.

In addition to the standard reactions described above, multiple controls were performed as follows: darkness controls were wrapped in aluminium foil; LPMO activity controls were without photopigments or without light exposure and were incubated with ascorbic acid and PASC; non-enzymatic oxidation controls of chlorophyllin, thylakoid suspensions, lignin and ascorbic acid were individually incubated with PASC and without LPMO enzymes under standard conditions (see above) to assess the possibility of spontaneous cellulose oxidation caused by light exposure (Fenton-type reactions).

### Sequential darkness or sunlight exposure

To verify the role of light as an inducer of cellulose oxidation via chlorophyllin, darkness and sunlight were sequentially applied to the same samples (results in Fig. 1d). The experiment was performed using the standard conditions for light-induced electron transfer (see above). The reaction mixture contained PASC, chlorophyllin, ascorbic acid and *Tt*LPMO9E with a lower dosage, using only 4 μg (instead of 10 μg) for a lower level of product release. The reaction mixtures were exposed to darkness for 2 h followed by sunlight exposure for 5 min. This cycle was subsequently repeated. After each change in light/darkness exposure, an aliquot was removed from the vials for further analysis. All aliquots were analysed by high-performance anion exchange chromatography (HPAEC) to measure the release of oligosaccharides and additionally treated with cellulase to quantify the monomeric glucose and gluconic acid (see above). An LPMO activity control, applying the same LPMO dosage was incubated for 24 h and afterwards treated with cellulase to quantify the monomeric glucose and gluconic acid (see above).

### Oxygen consumption measurements

The oxygen consumption was measured with a Chlorolab 2 System (Hansatech, England), with an oxygen sensor mounted at the bottom of a sealed reaction chamber. The 1 ml reaction chamber was stirred by a magnet and was equipped with a window for light exposure. Reactants could be added via a capillary cannula at the top, and no oxygen exchange with the external environment was observed. The measurements are given as oxygen concentration in μmol per ml. White LED light (4100 K) at 150 μmol photons per m^2^ per s was applied. The reactants were added at the beginning according to the dosages explained in the standard conditions, but at a lower temperature of 25 °C. Note that there is no headspace in the reaction chamber, why much less oxygen is available compared with the conditions described in standard conditions for enzymatic reactions. The combinations tested were: complete photosystem: LPMO (*Tt*LPMO9E)+chlorophyllin+Asc+PASC; the negative control experiments: chlorophyllin+Asc+PASC (no enzyme present); and the LPMO+Asc+PASC (no pigment present). From 0 to 900 s these reactions were carried out in darkness, while from 900 to 2,400 s white light was supplied (results shown in Fig. 2a).

The complete photosystem (LPMO+chlorophyllin+Asc+PASC) was measured with alternating cycles of light/darkness without or with the pigment. The conditions were identical to standard (see above) with the difference that *Tt*LPMO9E was dosed at only 0.01 mg ml^−1^ to lower the rate of oxygen consumption, and a temperature of 25 °C. *Tt*LPMO9E, ascorbic acid and PASC were first added. After five cycles of light and darkness (2 min each), chlorophyllin was added through the cannula, and three cycles of light and darkness were alternated (results shown in Fig. 2b).

### Lignin as reductant

Ascorbic acid, generally used as a reductant in the standard conditions for the enzymatic hydrolysis, was replaced with organosolv-extracted lignin to investigate its putative reducing capacity. The organosolv lignin was suspended in citrate-phosphate buffer (pH 6.3) and was added to a final concentration of 5 mg ml^−1^ (equal to a molar concentration of 25 mM based on lignin monomers) to the reaction mixture and incubated under standard conditions (see above) with *Tt*LPMO9E. Control experiments were performed as described above, replacing ascorbic acid with lignin.

### Light-induced oxidation of xyloglucans

Xyloglucan 1% w/w was used as a substrate, replacing PASC and incubated with chlorophyllin, ascorbic acid and *Tt*LPMO9E, the others parameters were according to standard conditions (see above). A release of oligosaccharides was detected using HAPEC. The observed pattern was a mixture of cello-oligosaccharides and xylogluco-oligosaccharides, which could not be fully separated, as seen for other published works^19^,^20^, therefore the peaks are separated into non-oxidized and oxidized products only.

### Light-induced oxidation of crystalline cellulose

Avicel was used as a cellulose substrate at 1% w/w instead of PASC and all other conditions were same as standard conditions (see above).

### Protein structure modelling

The PDB structures from the RCSB PDB Protein data bank were adapted for the HARLEM Molecular Modelling Package program (Kurnikov *et al*, available at http://harlem.chem.cmu.edu/). The PDB structures were (after adding hydrogens) analysed in HARLEM for possible LRET pathways from the surface to the metal ion^21^.

## Additional information

**How to cite this article:** Cannella, D. *et al*. Light-driven oxidation of polysaccharides by photosynthetic pigments and a metalloenzyme. *Nat. Commun.* 7:11134 doi: 10.1038/ncomms11134 (2016).

## Supplementary Material

Supplementary Information Supplementary Figures 1-9 and Supplementary Discussion
