# Supplementary material for: Light-driven oxidation of polysaccharides by photosynthetic pigments and a metalloenzyme
Source: Nat Commun. 2016 Apr 4;7:11134. doi: 10.1038/ncomms11134 (PMC4822002; doi:10.1038/ncomms11134)
Supplement: Supplementary Information — Supplementary Figures 1-9 and Supplementary Discussion [file ncomms11134-s1.pdf]

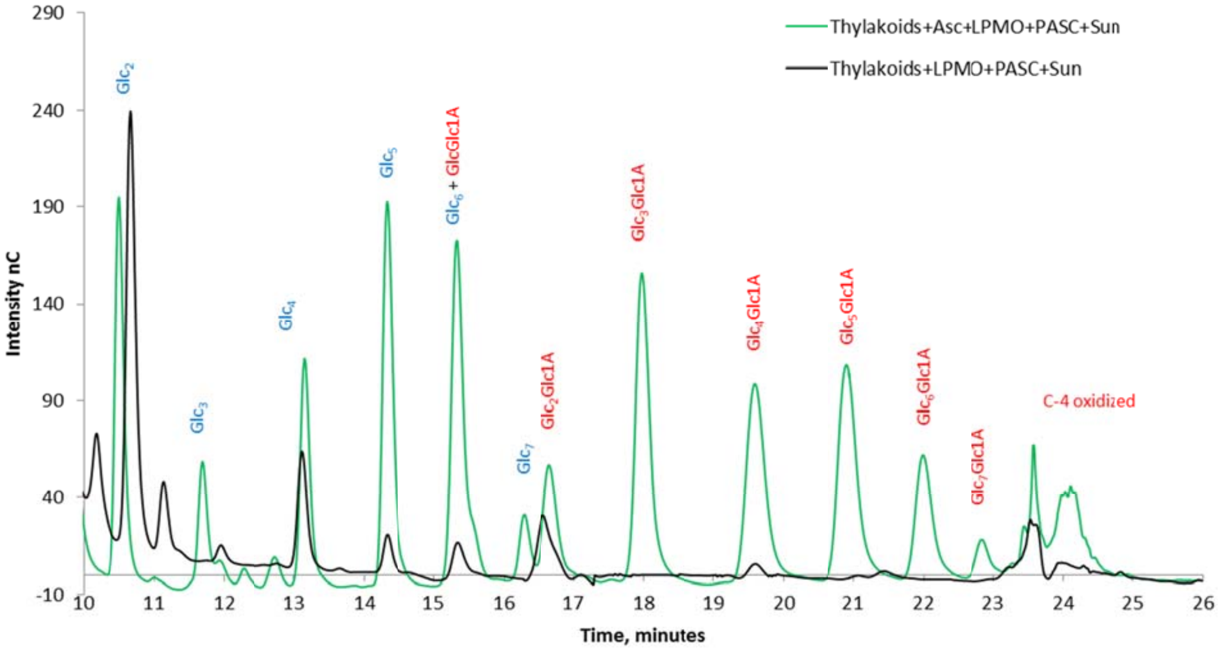

Supplementary Figure 1

Light-induced oxidation of PASC using thylakoid membranes from *A. thaliana*. The figure shows the HPAEC chromatographs of AA9 *Tt*LPMO9E, thylakoid membranes from *A. thaliana* with or without ascorbic acid after 3 hours of sunlight exposure. The experiment was performed as described in Methods section of the main text. Peak annotations of native oligosaccharides (blue) were done using the pure compounds as standard: Glc$_2$, cellobiose; Glc$_3$, cellotriose; Glc$_4$, cellotetraose; Glc$_5$, cellopentaose; Glc$_6$, cellohexaose; Glc$_7$, celloheptaose; oxidized oligosaccharides (red) were assigned by comparing with literature chromatograms performed with identical separation conditions as done by Westereng[32]: GlcGlc1A, cellobionic acid; Glc$_2$Glc1A, cellotrionic acid; Glc$_3$Glc1A, cellotetraonic acid; Glc$_4$Glc1A, cellopentaoinic acid; Glc$_5$Glc1A, cellohexaoinic acid; Glc$_6$Glc1A, celloeptaonic acid; Glc$_7$Glc1A, cellooctaonic acid. The chromatograms show the average of three independent experiments. The measured intensity of the signal in nC (nano Coulomb on the *y* axis) is without further adjustments.

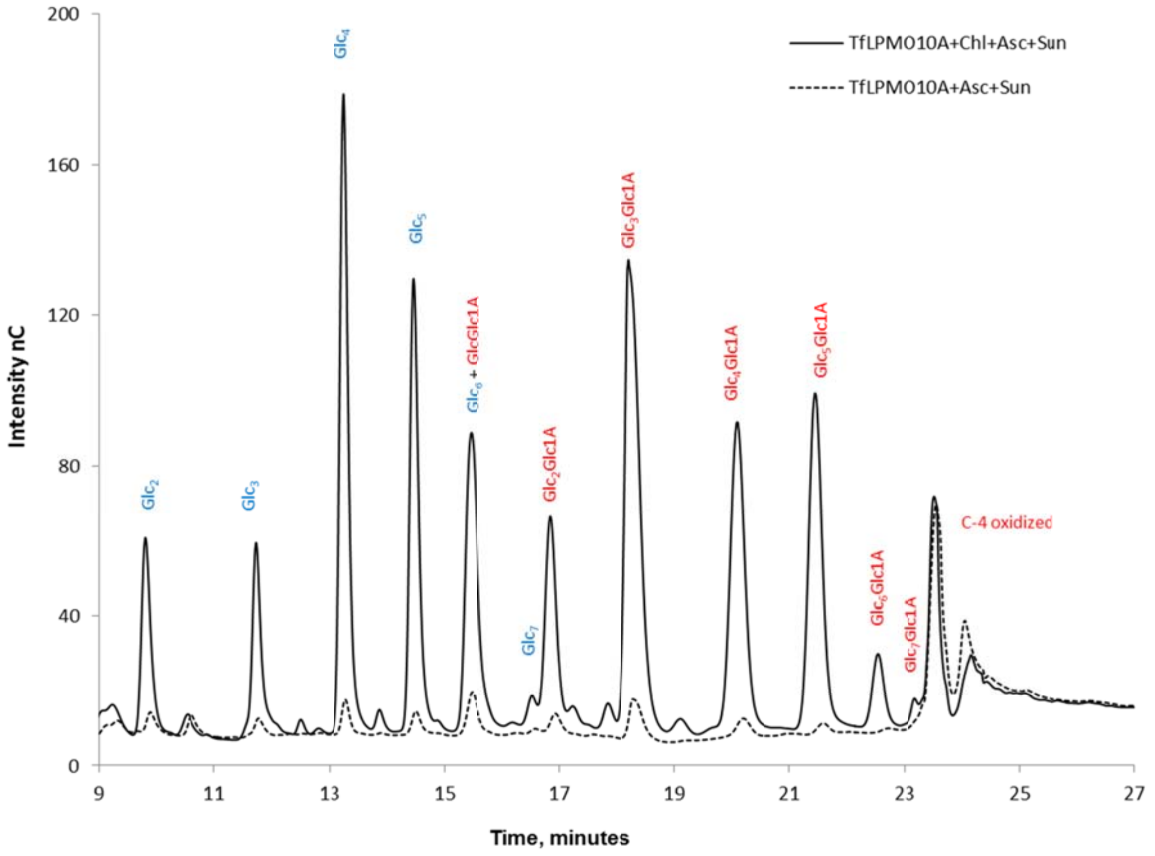

Supplementary Figure 2

Light-induced oxidation of PASC using AA10 *Tf*LPMO10A in combination with chlorophyllin, dioxygen, ascorbic acid and sunlight. The light-induced electron transfer system, or artificial photosystem, based on chlorophyllin as described for figure 1b (main paper) was also tested on bacterial AA10 LPMO. The figure shows the HPAEC chromatographs of LPMO from *Thermobifida fusca* after incubation with PASC, chlorophyllin and ascorbic acid exposed to sunlight (black chromatogram) for 3 hours. As a control, the LPMO was also incubated with PASC and ascorbic acid and exposed to sunlight (dotted chromatogram). Only after sunlight exposure PASC oxidation was detected using *Tf*LPMO10A. Peak annotations are the same as in Supplementary Figure 1.

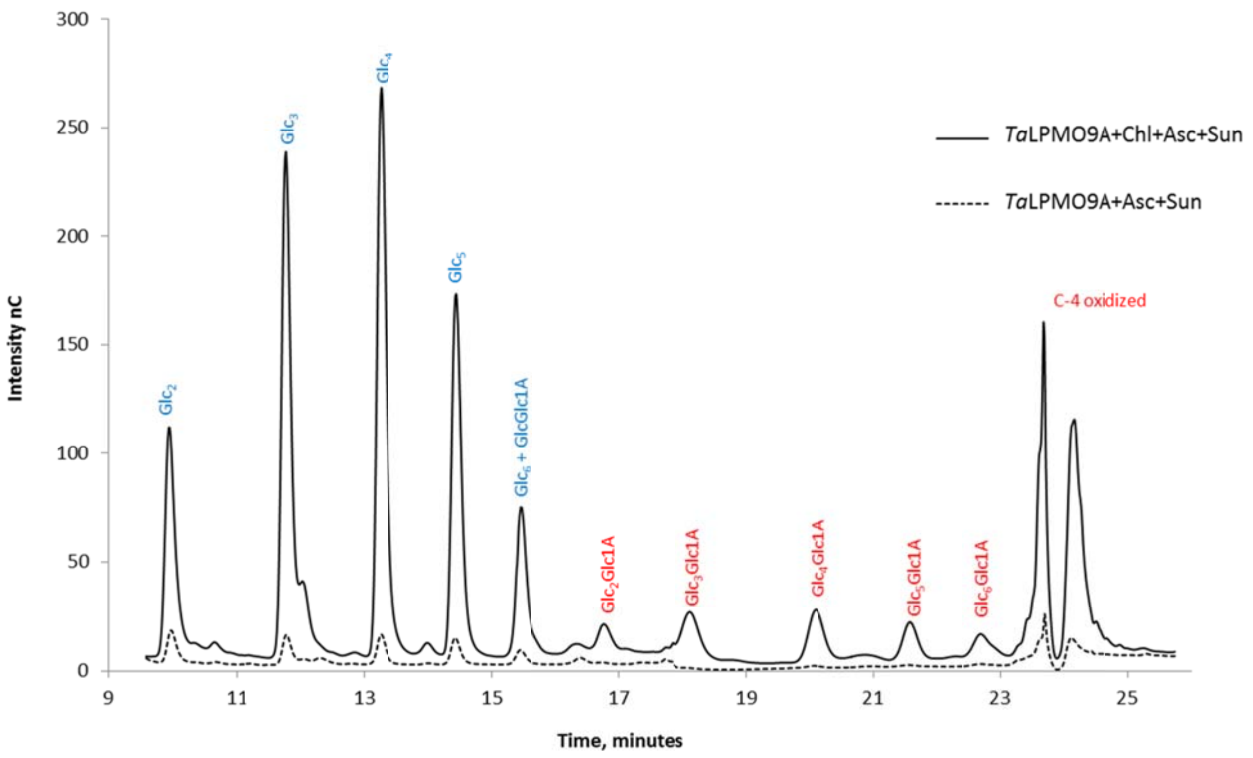

Supplementary Figure 3

Light-induced oxidation of PASC applying AA9 *Ta*LPMO9A in combination with chlorophyllin, ascorbic acid and sunlight. The figure shows the HPAEC chromatographs of AA9 *Ta*LPMO9A after incubation with PASC, chlorophyllin and ascorbic acid and irradiated by sunlight for 3 hours (black chromatogram). A control experiment was performed without chlorophyllin (dotted chromatogram). The results show light-induced cleavage of PASC by *TaLPMO9A* combined with chlorophyllin and ascorbic acid. The products released from *Ta*LPMO9A activity are predominantly cellooligomers but also the oxidized oligosaccharides in the form of aldonic acid and a range of unresolved C4 gem-diol oxidized oligosaccharides (in a non-resolved peak at the minute 24th are detected). The level of non-oxidized oligosaccharides production is in the same range as those obtained with *Tt*LPMO9E.

Peak annotations are the same as in Supplementary Figure 1.

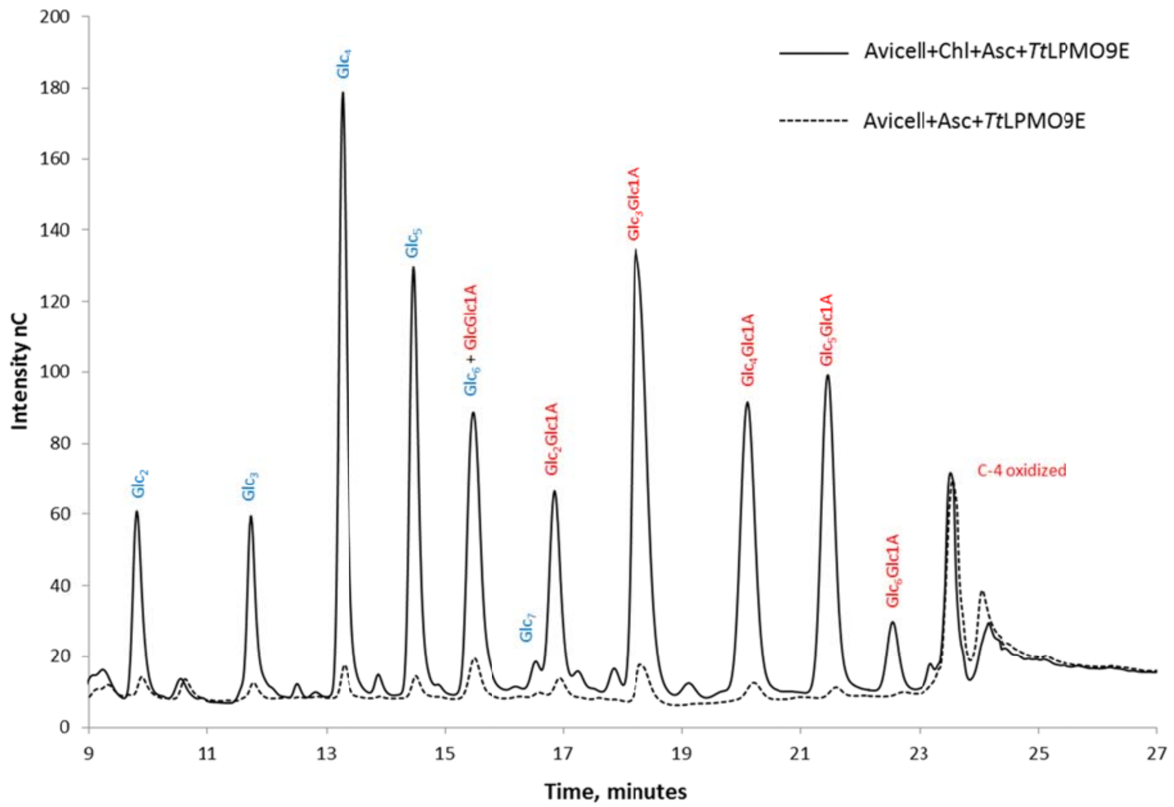

Supplementary Figure 4

The figure shows the HPAEC chromatographs of AA9 *Tt*LPMO9E after incubation with crystalline cellulose substrate (Avicel), chlorophyllin and ascorbic acid exposed to sunlight (black chromatogram). The control experiment without chlorophyllin (dotted chromatogram). Avicel was dosed at 1% w/w and all other conditions are equal to the standard conditions explained in Methods section of the main text. Peak annotations are the same as in Supplementary Figure 1.

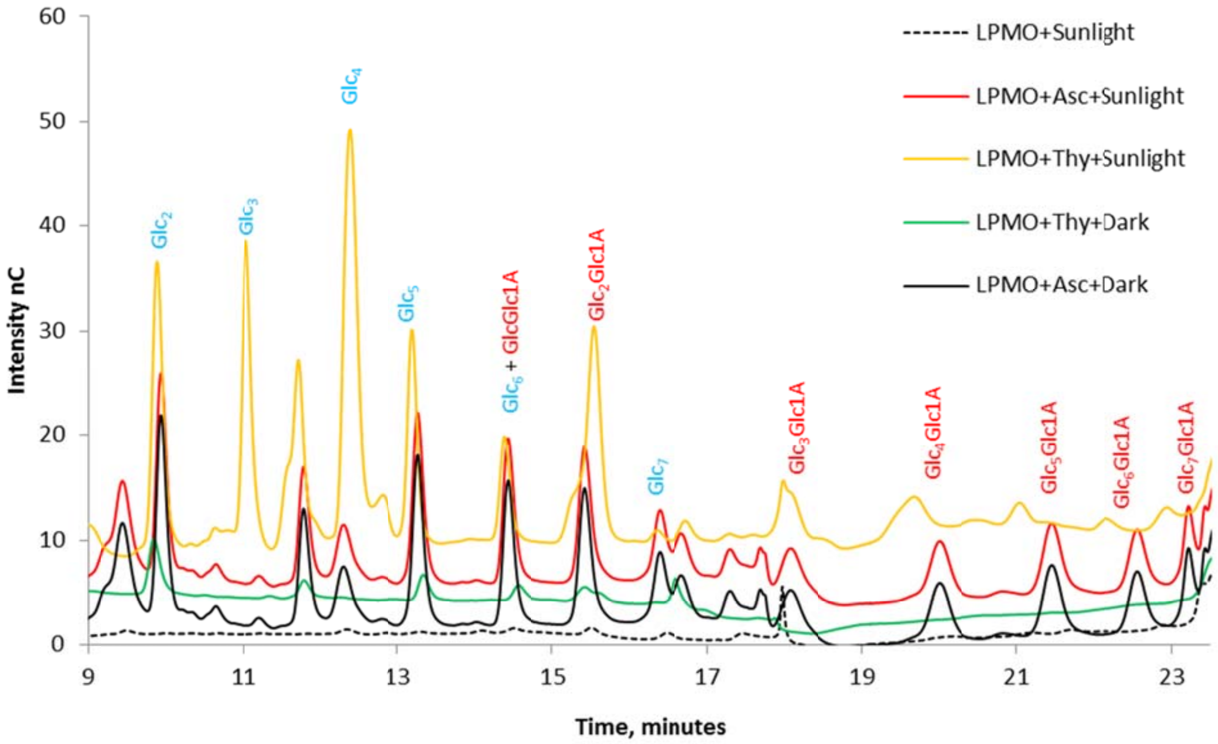

Supplementary Figure 5

Light-induced PASC oxidation by LPMO and a thylakoid suspension in the absence of the reductant ascorbic acid. The figure shows the HPAEC chromatographs of PASC after 3 hours incubation with *Tt*LPMO9E and a thylakoid suspension isolated from a cyanobacterium. As light source sunlight was applied and compared to darkness (yellow and green chromatograms, respectively). As control experiments PASC was incubated with ascorbic acid and LPMO but without thylakoid suspension (red chromatogram for sunlight and black chromatogram for darkness), or with LPMO but without thylakoid suspension or ascorbic acid (dotted chromatogram) and exposed to sunlight. Peak annotations are the same as in Supplementary Figure 1.The measured intensity of the signal in nC (nano Coulomb on the *y* axis) is without further adjustments.

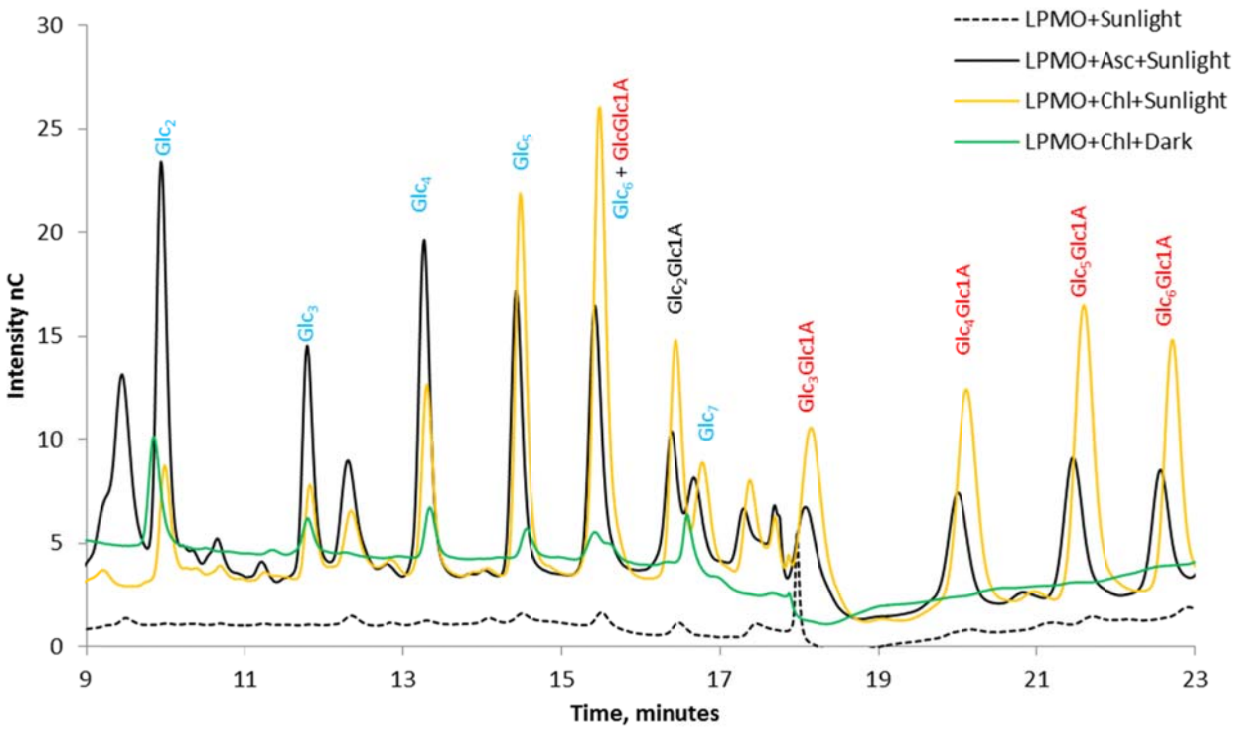

Supplementary Figure 6

Light-induced PASC oxidation by LPMO and chlorophyllin in the absence of the reductant ascorbic acid**.** The figure shows the HPAEC chromatographs of PASC oxidation after incubation with *Tt*LPMO9 and chlorophyllin. As light source sunlight was applied and compared to darkness (yellow and green chromatogram, respectively). As control experiments PASC was incubated with ascorbic acid and LPMO (black chromatogram), or with LPMO but without chlorophyllin and ascorbic acid (dotted chromatogram). Peak annotations are the same as in Supplementary Figure 1. The measured intensity of the signal in nC (nano Coulomb on the *y* axis) is without further adjustments.

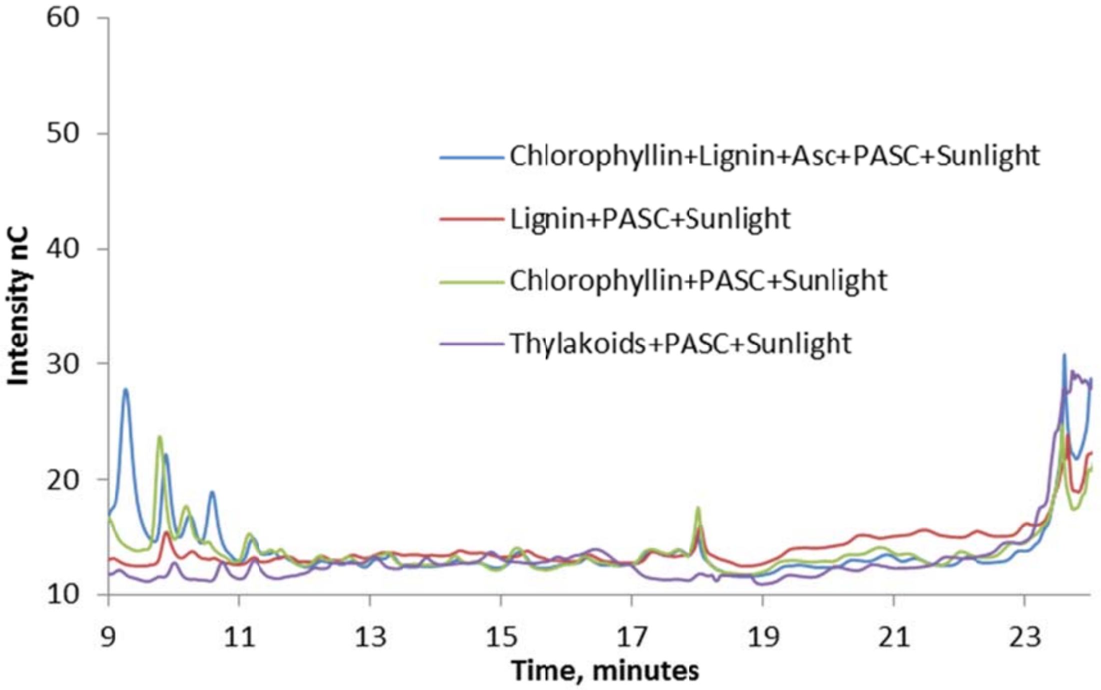

Supplementary Figure 7.

Control experiments without LPMO enzyme. To test the possibility of oxidation products from reactions caused by ROS generated by light exposure of the pigments, controls without LPMO enzyme were performed. Chlorophyllin, thylakoids and lignin were incubated with PASC, as single components or combined with ascorbic acid and were exposed to sunlight for 3 hours under standard conditions, described in Methods section of the main text. The samples were measured by HPAEC to detect any PASC breakdown products. No light dependent PASC oxidation products were detectable which indicates that the light-induced PASC oxidation is not caused by non-enzymatic, i.e. Fenton-type reactions in solution. This confirms that the PASC oxidation products shown in Figure 1 of the main text are the results of a LPMO catalyzed reaction. The experimental conditions were applied as described in Methods section of the main text, but without LPMO enzyme. Degradation products from PASC were not detected. Peaks at 9 - 11 min are chlorophyllin and lignin; peaks at 18 min are caused by the pump gradient; peaks from 23 min are ascorbic acid or thylakoids.

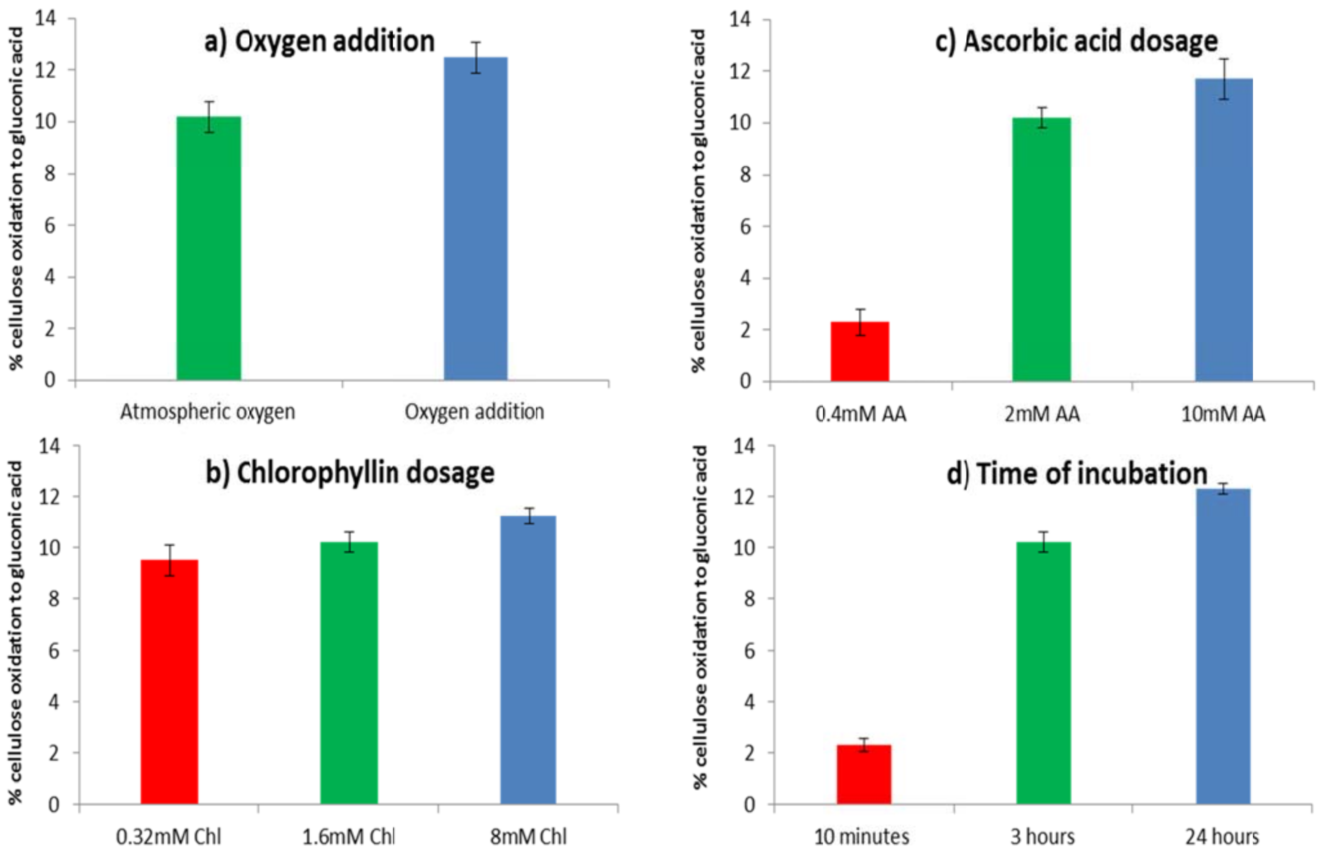

Supplementary Figure 8

Optimization of reaction parameters. a) Oxygen addition: oxidation of PASC with *Tt*LPMO9E, chlorophyllin and ascorbic acid at elevated and atmospheric levels of dioxygen in the vial headspace. Atmospheric level (green) and pure dioxygen gas (blue). b) Chlorophylln dosage: oxidation of PASC with *Tt*LPMO9E, chlorophyllin and ascorbic acid at different levels of chlorophyllin: 0.32 mM chlorophyllin (red), 2 mM chlorophyllin (green), 8 mM chlorophyllin (blue). c) Acid ascorbic dosage: oxidation of PASC with *Tt*LPMO9E and chlorophyllin at different levels of ascorbic acid. 0.4 mM ascorbic acid (red), 2mM ascorbic acid (green), 10 mM ascorbic acid (blue). d) Time of incubation: light induced oxidation of PASC cellulose at varying length of sunlight exposure using chlorophyllin as photopigment, ascorbic acid and *Tt*LPMO9E. The bars shows respectively 10 minutes (red), 3 hours (green) and 24 hours (blue) of sunlight exposure. The experiment was conducted as described in Methods section of the main text.

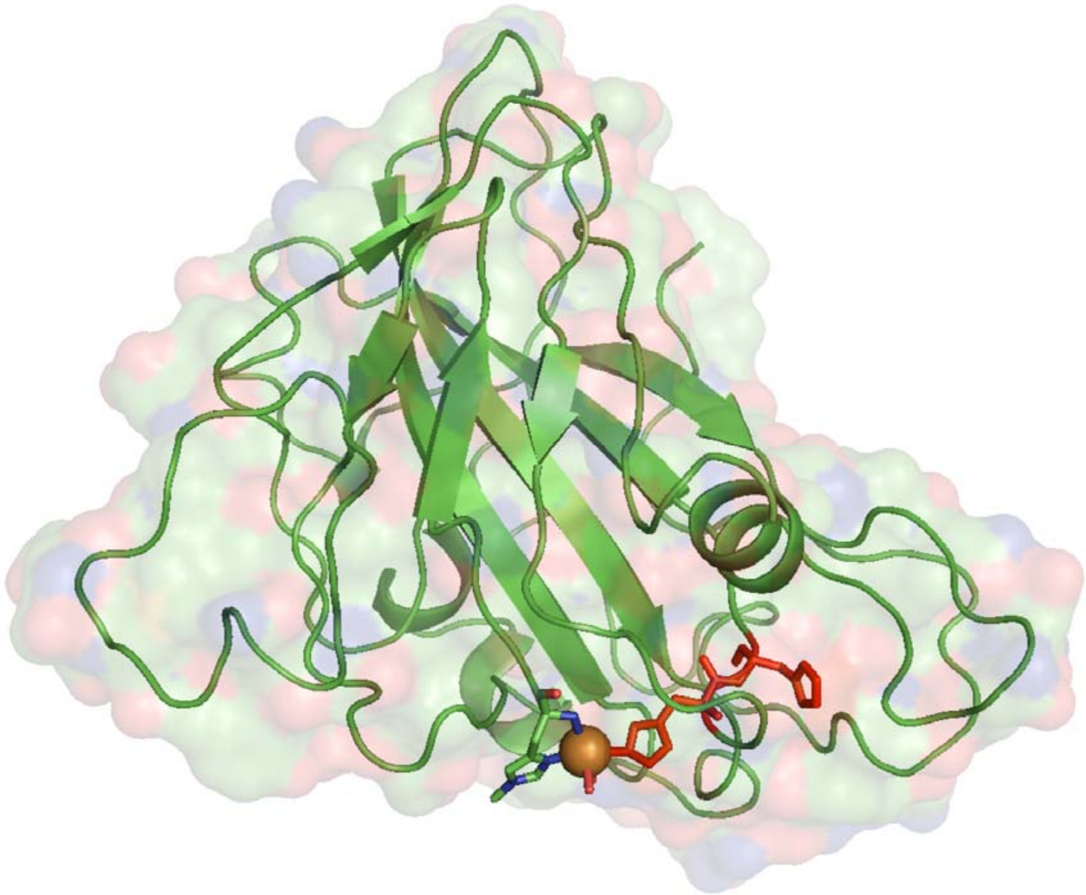

Supplementary Figure 9. The structure (PDB ID: 2YET) of *Thermoascus aurantiacus* LPMO (*Tt*LPMO9E) showing (in red) a possible static pathway from His87 to Cu(II).

The search for simple static pathways in known structures of the AA9 family was conducted using the Molecular Modeling Package HARLEM (http://harlem.chem.cmu.edu) based on the pathway model proposed by Onuchic and Beratan[19]. The figure shows the pathway as a stick structure from His87 through His86 to Cu(II) through 12 covalent bonds equivalent to a tunneling length of 16.8 Å with a calculated pathway coupling decay value of 2.2 10^{-3} from His87 NE2 to Cu(II). His87 is sitting on the side of LPMO and is thus free of cellulose that is binding to LPMO during catalysis. In another structure of the same enzyme soaked in 10 mM $Cu(NO_3)_2$ (PDB 3ZUD) a Cu(II) ion is found coordinating to this His87 supporting that it is a solvent exposed amino acid.[5] A search conducted on other structures in the AA9 family revealed that similar pathways could be found.

They are all located very much in the same position but with different ligands at the surface *Thermoascus auranticus(* PDB 2YET): NE2-His87His86-Cu; *Neurospora crassa* (PDB 4EIR): NZ-Lys85His84-Cu; *Neurospora crassa* (PDB 4D7U): NZ-Lys84His83-Zn); *Trichoderma reesei* (PDB 2VTC): HO-Tyr91Pro90His89-Ni.

<u>Supplementary Discussion</u>

Light-induced effects of the individual assay components

Detailed HPAEC chromatograms of PASC oxidation by *T. terrestris* LPMO or thylakoids or chlorophyllin exposed to sunlight and compared to a control containing only LPMO and ascorbic acid are shown in Supplementary Figures 5 and 6.

The ability of pigments to harvest light and transfer electrons upon excitation was first observed having LPMO incubated with pigments only (thylakoids or chlorophyllin, Supplementary Figure 5 and 6 respectively) and PASC, without reductant (ascorbic acid). The reaction mixtures were incubated in sunlight or in darkness, showing PASC oxidation only when the mixture was exposed to sunlight (yellow line). Furthermore, as negative control, LPMO was incubated with PASC and exposed to sunlight.

Supplementary Figure 6 shows, as a positive control experiment, the activity of LPMO when incubated with ascorbic acid and PASC, exposed to sunlight (red line); same experiment conducted in darkness resulted in the same level of PASC oxidation and profile of oxidized products (black line). There is no indication of a light-induced response by incubation of LPMO and ascorbic acid. For the sunlight exposed LPMO, no PASC oxidation products were detected, neither was there any reaction for LPMO and pigments in darkness (Supplementary Figure 5 and 6, dotted and green line,

respectively). The incubation parameters and reactant dosages were chosen accordingly to the standard conditions described in Methods section of the main text.

The overall amount of PASC oxidation with the pigments and enzyme only is about 10 times lower than what is observed for the full light-induced system including a reductant (Fig. 1a,b main paper). Without the reductant the pigments are degraded by photobleaching. The distribution between oxidized and non-oxidized cello-oligomers is different for thylakoids and chlorophyllin, however, this difference disappears when the reductant is added and the artificial photosystem is complete, see Figure 1a and 1b. Further experimental details are described in Methods section of the main text.

Optimization of reaction parameters

To test that the reaction was not rate-limited, the rate of oxygen consumption for the sequential experiment having different amounts of initial dissolved oxygen (respectively 0.31, 0.29 and 0.27 μmol mL^{-1}), was measured to be constant at 0.12±0.005 nmol $mL^{-1}s^{-1}$, see figure 2b main text. Below is described in details the optimization of the reaction parameters evaluated in terms of final amount of cellulose oxidation in percentage of the initial amount of substrate. Dioxygen is needed for LPMO catalysis, and if not available in sufficient amount it may be limiting for the oxidation of the cellulose. In the headspace and liquid a total of 14.3 μmol dioxygen is present at 50 ^{o}C. The measured oxidation of 10% of the PASC (Fig. 1c) requires 0.56 μmol of dioxygen, thus oxygen is in excess compared to the observed level of oxidation.

The effect of increased oxygen concentration was tested by supplementing pure dioxygen gas in the 1.8 mL head space of the reaction vial just before the start of the reaction. As shown in Supplementary Figure 8a after 3 hours, the level of oxidation was on average 15% higher when 5 times more oxygen (blue bar) was supplied compared to the standard condition with atmospheric air

(green bar). However, the oligosaccharide amounts were identical when the incubation was prolonged to 24 hours (data not shown). This shows that the dioxygen level present in the samples was sufficient for the assays performed. This result further supports that a putative oxygen production by thylakoids upon light exposure was not the cause of the increased level of oxidation of cellulose observed in Fig.1a.

Different dosages of chlorophyllin were tested with 0.32, 1.6 and 8 mM chlorophyllin in the final reaction volume. 3 hours of sunlight exposure and standard conditions were applied as described in Methods section of the main text. Less than 5% increase in oxidized cellulose was observed comparing 0.32 to 1.6 mM and a 15% higher level of oxidized cellulose was observed comparing 1.6 mM to 8 mM of chlorophyllin. 1.6 mM was therefore chosen as the standard concentration of chlorophyllin.

Ascorbic acid was applied in three different amounts: 0.4, 2 and 10 mM together with chlorophyllin at 1.6 mM and *Tt*LPMO9E. All samples were exposed to sunlight for 3 hours. The increase of oligosaccharides production was proportionally correlated with the ascorbic acid molarity, when comparing 0.4 to 2 mM of ascorbic acid. However, only a 20% increase of oxidation products was achieved by increasing the amount of ascorbic acid from 2 mM to 10 mM. Thus 2 mM was chosen as the standard dosage of ascorbic acid.

The light-induced electron transfer based on chlorophyllin and the standard conditions (described in Methods section of the main text) was performed for 10 minutes, 3 and 24 hours. In supplementary figure 8d shows that within 10 minutes 2.3% of cellulose was, whereas 10.2 % and 12.3% of cellulose oxidation within 3 and 24 hours respectively.
